# Effect of OH^−^ on chemical mechanical polishing of β-Ga_2_O_3_ (100) substrate using an alkaline slurry

**DOI:** 10.1039/c7ra11570a

**Published:** 2018-02-08

**Authors:** Chuanjin Huang, Wenxiang Mu, Hai Zhou, Yongwei Zhu, Xiaoming Xu, Zhitai Jia, Lei Zheng, Xutang Tao

**Affiliations:** College of Mechanical and Electrical Engineering, Nanjing University of Aeronautics and Astronautics Nanjing 210016 China meeywzhu@nuaa.edu.cn; College of Mechanical Engineering, Yancheng Institute of Technology Yancheng 224051 China; State Key Laboratory of Crystal Materials, Key Laboratory of Functional Crystal Materials and Device, Shandong University Jinan 250100 China txt@sdu.edu.cn

## Abstract

β-Ga_2_O_3_, a semiconductor material, has attracted considerable attention given its potential applications in high-power devices, such as high-performance field-effect transistors. For decades, β-Ga_2_O_3_ has been processed through chemical mechanical polishing (CMP). Nevertheless, the understanding of the effect of OH^−^ on β-Ga_2_O_3_ processed through CMP with an alkaline slurry remains limited. In this study, β-Ga_2_O_3_ substrates were successively subjected to mechanical polishing (MP), CMP and etching. Then, to investigate the changes that occurred on the surfaces of the samples, samples were characterised through atomic force microscopy (AFM), three-dimensional laser scanning confocal microscopy (LSCM), scanning electron microscopy (SEM) and X-ray photoelectron spectroscopy (XPS). LSCM and SEM results showed that β-Ga_2_O_3_ is highly vulnerable to brittle fracture during MP. AFM revealed that an ultrasmooth and nondamaged surface with a low *R*_a_ of approximately 0.18 nm could be obtained through CMP. XPS results indicated that a metamorphic layer, which mainly contains soluble gallium salt (Ga(OH)_4_^−^), formed on the β-Ga_2_O_3_ surface through a chemical reaction. A dendritic pattern appeared on the surface of β-Ga_2_O_3_ after chemical etching. This phenomenon indicated that the chemical reaction on the β-Ga_2_O_3_ surface occurred in a nonuniform and selective manner. The results of this study will aid the optimization of slurry preparation and CMP.

## Abstract 2

β-Ga_2_O_3_, a semiconductor material, has attracted considerable attention given its potential applications in high-power devices, such as high-performance field-effect transistors.

## Graphical abstract

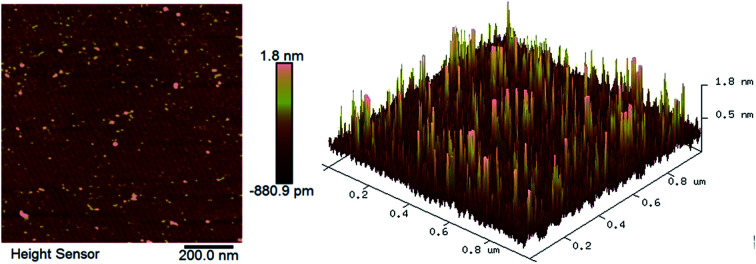

## 1 Introduction

The application of β-type gallium oxide (β-Ga_2_O_3_) as a semiconductor material of high-power devices,^1–3^ such as high-performance field-effect transistors,^4,5^ Schottky barrier diodes^6,7^ and ultraviolet transparent electrodes,^8^ has attracted considerable attention because β-Ga_2_O_3_ has excellent material properties, including a wide band gap of approximately 4.9 eV, and stable chemical and thermal properties.^9^ Although numerous studies have been conducted on β-Ga_2_O_3_ materials,^10–14^ few have focused on the ultraprecision machining of these materials. Chemical mechanical polishing (CMP), a key step in the ultraprecision machining of crystal materials, is an efficient technique that yields ultrasmooth and undamaged crystal surfaces.^15^

The main cleavage plane of the β-Ga_2_O_3_ crystal is along its (100) surface,^16^ which is the most thermodynamically stable and the most suitable surface for thin-film growth among all surfaces.^17^ However, cleavage renders the crystal surface fragile and hinders the ultraprecise machining of β-Ga_2_O_3_.^2^ Ga–O bonds in the unstable octahedral of the β-Ga_2_O_3_ (100) structure fracture easily.^16^ The β-Ga_2_O_3_ (100) structure is composed of a slightly distorted tetrahedral and a highly distorted octahedral. Ga–O bonds in β-Ga_2_O_3_ (100) are highly sensitive to mechanical stress and cause crystal cleavage during processing. Therefore, chemical action may be a feasible method for generating new bonds that can shield Ga–O bonds during CMP.^18–20^ Slurries with alkali and oxidisers applied in the CMP of GaN and GaAs can be applied in the CMP of β-Ga_2_O_3_.^18,20,21^ However, gallium oxide is in a stable oxidation state, and wet etching experiments on β-Ga_2_O_3_ are utilised only as a reference for evaluating the use of sodium hydroxide as a mordant without an oxidant.^22,23^ The β-Ga_2_O_3_ (100) surface needs to be processed through CMP using an alkaline slurry with OH^−^ to obtain an ultrasmooth crystal surface. Moreover, the chemical action mechanism that underlies the CMP of β-Ga_2_O_3_ must be investigated.

By evaluating the morphology and chemical composition of the β-Ga_2_O_3_ surface after treatment, this study investigated the effect of OH^−^ on β-Ga_2_O_3_ processed through mechanical polishing (MP) without chemical action and to CMP with chemical action. Moreover, the chemical action between β-Ga_2_O_3_ and sodium hydroxide during wet etching was investigated. The surface morphology of β-Ga_2_O_3_ was examined through atomic force microscopy (AFM), laser-scanning confocal microscopy (LSCM) and scanning electron microscopy (SEM). Results indicated that a metamorphic layer that formed on the crystal surface through a chemical reaction was removed during CMP. X-ray photoelectron spectroscopy (XPS) was also used to study the surface chemistry of β-Ga_2_O_3_. The different chemical states of the metamorphic layer during different treatment processes were studied. This experimental analysis may be useful for optimising slurry preparation.

## 2 Material and methods

β-Ga_2_O_3_ substrates were cut from a crystal bar along the (100) plane and prepared *via* the edge-defined film-fed crystal growth method. The β-Ga_2_O_3_ (100) substrates had a size of approximately 10 mm × 10 mm × 1 mm (length × width × thickness). Prior to the experiments, the substrates were ground to expose a large flat surface, which served as the foundation of subsequent experiments.

This experiment comprised three steps, as follows: firstly, the substrates were treated through MP. β-Ga_2_O_3_ was polished using a polishing tester (UNIPOL-1502, MTI-KJ Corp., Ltd.) with silk as a soft pad at a pressure of 15 kPa. Deionised water (DI) and diamond paste (mean diameter 1 μm) were used as chemically inert lapping media. The other process conditions were as follows: plate speed of 35 rpm and polishing time of 4 h. Secondly, the substrates were treated through CMP. β-Ga_2_O_3_ was polished with the same polishing tester equipped with an Politex pad (Rohm & Haas Co., Ltd.). Commercial colloidal silica (NS-54, Fuso Chemical Co., Ltd.) mixed with diluted sodium hydroxide was used as the slurry. Slurry pH was adjusted to 11, and slurry concentration was approximately 20 wt%. The slurry was not reused. The other process conditions were as follows: plate rotation speed of 35 rpm, operating pressure of 15 kPa, slurry flow rate of 50 mL min^−1^ and polishing time of 15 h. Finally, the β-Ga_2_O_3_ substrates were etched with 20 wt% sodium hydroxide solution at 55 °C for 2 h. The substrates were successively cleaned with liquid cleaner, DI water and ethyl alcohol and dried by an air spray gun for each measurement.

The surface morphology of β-Ga_2_O_3_ after treatment was characterised using Keyence VK-X110K 3D LSCM, FEI Nova NanoSEM 450 and Bruker Dimension Icon AFM. The surface average roughness (*R*_a_) values after MP were determined using LSCM with 100 μm × 100 μm scans. *R*_a_ after CMP was determined through AFM with 1 μm × 1 μm scans. The surface chemical composition was analysed with Thermo Scientific Escalab250Xi (XPS). Firstly, samples subjected to MP, CMP and etching were studied. The surfaces of these samples were measured in normal mode with 0° electron emission angle. Secondly, to analyse the chemical composition of the crystal surface subjected to CMP, the crystal surface was measured in angle-resolved mode with an electron emission angle of 0° to 75°. All data were obtained using a monochromatised Al-Kα source (1486.6 eV) and a pass energy of 40 eV. XPS peak assignment was based on an acceptable standard reference database and literature reports.^17,24–26^

## 3 Results and discussion

### 3.1 Surface morphology analysis

**Fig. 1:**
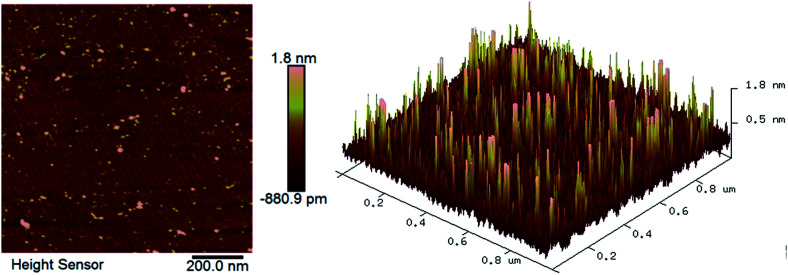
AFM images of β-Ga_2_O_3_ after CMP.

The AFM images of the CMP-treated β-Ga_2_O_3_ surface are shown in Fig. 1. The *R*_a_ of the sample was approximately 0.18 nm. The surface did not exhibit a regular terraced morphology but instead exhibited negligible roughness similar to that reported in previous studies.^27,28^ This result indicated that the wafer surface might have been covered with a layer of chemical substances. The ultrasmooth surface of the wafer processed through CMP is shown in Fig. 1. The wafer exhibited surface contamination, which is shown as a series of brightly coloured points.^29^ The properties of the polished surface with residual chemical products were characterised through SEM and XPS to identify the chemical action mechanism that underlies the CMP of β-Ga_2_O_3_.

**Fig. 2:**
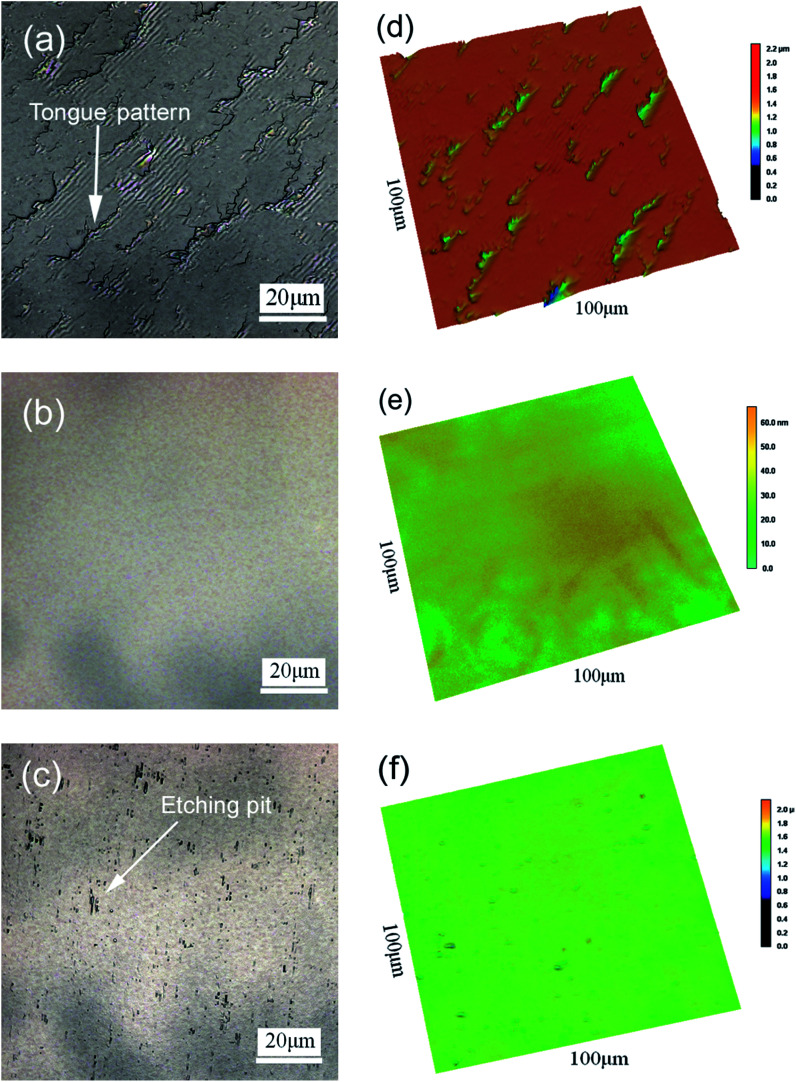
Two-dimensional LSCM images of β-Ga_2_O_3_ (100) after (a) MP, (b) CMP and (c) etching. (d), (e) and (f) are the corresponding three-dimensional images of (a), (b) and (c).

The surface morphology of β-Ga_2_O_3_ treated through MP, CMP and etching are shown in Fig. 2. As shown in Fig. 2(a) and (d), after MP treatment, the substrate appeared rough with an *R*_a_ of 52 nm and was covered with tongue-patterned cleavage pits. During MP, abrasive diamond grains cut into the crystal plane and resulted in the instantaneous generation of concentrated stress. New cracks were initiated on the crystal plane, and crack expansion accelerated until the plane underwent low-energy brittle fracture, which is very easy to occur because of the cleavage of β-Ga_2_O_3_. After CMP, the surface *R*_a_ of the β-Ga_2_O_3_ substrate decreased from 54 nm to 0.18 nm, and the cleavage pits disappeared. The ultrasmooth mirror-like surface of the substrate obtained after CMP is shown in Fig. 2(b) and (e). Micro cleavage pits caused by local cleavage fracture were difficult to remove during MP, as shown in Fig. 2(a) and (b). By contrast, micro cleavage pits were easily removed during CMP because β-Ga_2_O_3_ was chemically corroded in the alkaline environment. Numerous etching pits were present on the surface of β-Ga_2_O_3_, as shown in Fig. 2(c) and (f). This pattern indicated that a chemical reaction occurred between Ga_2_O_3_ and sodium hydroxide. The surface morphology of the β-Ga_2_O_3_ substrate dramatically changed through chemical action.

**Fig. 3:**
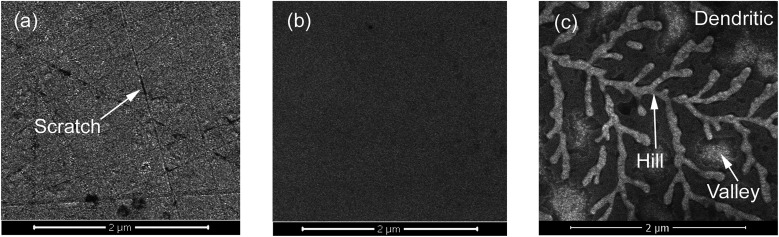
SEM images of the β-Ga_2_O_3_ surface after (a) MP, (b) CMP and (c) etching.

The crystal surfaces obtained through different processes using slurries containing sodium hydroxide were subjected to microscopy observation to describe the chemical mechanism that acts on β-Ga_2_O_3_ during CMP. The SEM images of the surfaces treated through MP, CMP and etching are shown in Fig. 3. As shown in Fig. 3(a), numerous scratches formed on the crystal surface after MP. Scratches between cleavage pits appeared flat, as shown in Fig. 2(a). The crisscrossing microscratches indicated that the cleavage properties of β-Ga_2_O_3_ caused microscratch defects even when abrasive grains did not produce cleavage pits after the crystal face was cut. An ultrasmooth mirror-like surface was obtained after CMP, as shown in Fig. 3(b). This surface exhibited fewer cleavage defects than the surface shown in Fig. 3(a). Fig. 3(c) shows the β-Ga_2_O_3_ surface after etching with sodium hydroxide solution and CMP. The crystal face exhibited a dendritic pattern. The bright section of the dendritic pattern shown in Fig. 3(c) represented a protruding structure similar to a peak, and the remaining grey portion indicated a low-lying area similar to a valley. The presence of dendrites implied that sodium hydroxide selectively corrodes β-Ga_2_O_3_. The valleys of the crystal surface indicated areas that are susceptible to corrosion under alkaline conditions, and the peaks indicated areas that are relatively resistant to corrosion. Generally, β-Ga_2_O_3_ exhibits uneven corrosion under alkaline conditions during CMP. Therefore, the use of abrasive silica particles to remove the residual metamorphic layer is an essential step in CMP.

The formation and removal of the metamorphic layer on the surface of β-Ga_2_O_3_ during CMP are important for obtaining an ultrasmooth surface. The chemical composition of the metamorphic layer was analysed.

### 3.2 Surface chemical composition analysis

**Fig. 4:**
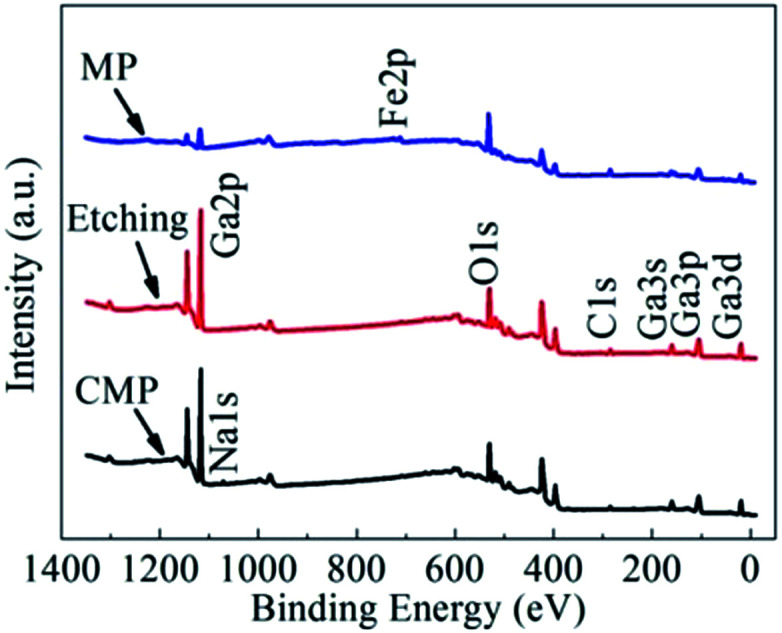
Wide-survey XPS spectra of β-Ga_2_O_3_ after CMP, etching and MP.

As shown in Fig. 4, peaks that correspond to Ga 3d, O 1s and C 1s photoelectrons are present in the survey scan spectra of the β-Ga_2_O_3_ substrates treated through MP, CMP and etching. The prominent C 1s peaks observed in all wide-survey XPS spectra may have originated from the adsorption of adventitious carbon contaminants on the surface; these contaminants could be partly removed by heat treatment.^17^ The C 1s peak at 284.8 eV could be used to calibrate the peak positions of other elements. Fig. 4 also shows that surface contaminants can be attributed to different processes. For example, Fe was contributed by the iron plate during grinding, and Na was contributed by the slurry used for CMP. These contaminants could be removed through subsequent processes. The spectra of the surfaces would be similar if the effect of Fe and Na contaminants are ignored.

**Fig. 5:**
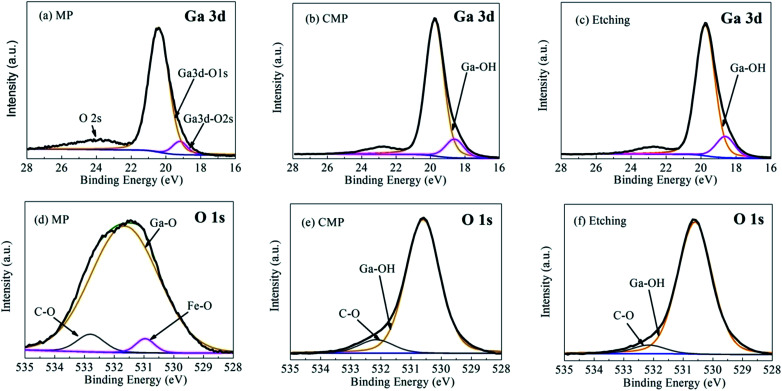
(a)–(f) Fitted Ga 3d and O 1s XPS spectra of β-Ga_2_O_3_ after MP, CMP and etching.

Fig. 5 shows the Ga 3d and O 1s XPS spectra of the β-Ga_2_O_3_ surface after various treatments. Curve fitting was performed by using the Shirley background, and the symmetric Lorentzian–Gaussian peak shape function value was set as 20%. The evolution of the Ga 3d XPS spectra of the β-Ga_2_O_3_ surface after different treatments is illustrated in Fig. 5(a)–(c). Fig. 5(a) shows that the peak representing the Ga–O bond of β-Ga_2_O_3_ is located at 20.4 eV after MP, whereas that representing O 2s, which could be attributed to the hybridization of the Ga 3d and O 2s surface states, is located at 24 eV.^24^Fig. 5(a)–5(b) show that the Ga 3d peak acquired at normal emission shifted to a low binding energy from MP to CMP. This shift was caused by a chemical reaction. After CMP, the peak position shifted by approximately 0.9 eV from 20.4 eV (Ga–O bond) to 19.5 eV (Ga–OH bond).^26^ This shift could be attributed to the breakage of Ga–O bonds through a tribochemical action, which allowed surface Ga to bond with hydroxyl from sodium hydroxide. This result suggested that the surface of β-Ga_2_O_3_ undergoes tribochemical reactions with sodium hydroxide to form new reaction products, such as soluble gallium salt (Ga(OH)_4_^−^), during CMP.^18^ These salts were then partly absorbed on the polished surface. Chemical reactions during CMP and etching exhibited similar results, as shown in Fig. 5(c). A detailed analysis of the O 1s core level peaks of the substrate is shown in Fig. 5(d)–(f). The O 1s peaks at 531.4, 532.8 and 530.9 eV observed after MP and shown in Fig. 5(d) could be respectively attributed to the main Ga–O bond peak,^24^ C–O from carbon contaminants^17^ and Fe–O contributed by cast-iron grinding discs before MP.^26^Fig. 5(d)–(f) show that the Ga–O bond of β-Ga_2_O_3_ was replaced by Ga–OH after CMP and etching due to chemical action, indicating that the same condition shown by Fig. 5(a)–(c) was observed for CMP. Moreover, the low full width at half maximum (FWHM) of the Ga 3d and O 1s peaks observed from Fig. 5(b) and (e) indicated the excellent crystalline quality of the studied samples.^9^

**Fig. 6:**
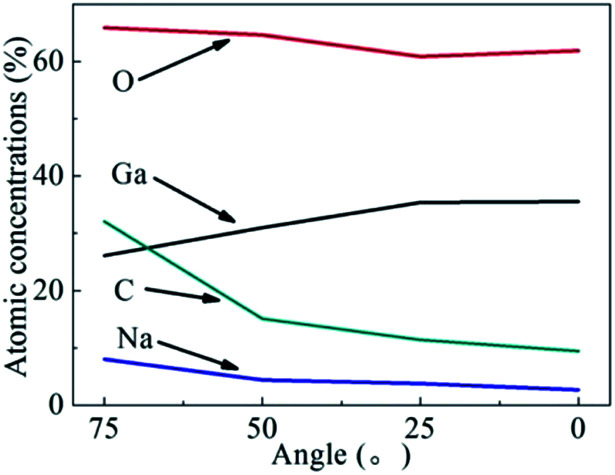
ARXPS results of the β-Ga_2_O_3_ surface layer treated through CMP.

Fig. 6 shows the variation tendency of the atomic composition, including Ga, O, C and Na, of the β-Ga_2_O_3_ surface layer after CMP. The atomic composition of the β-Ga_2_O_3_ surface layer was identified through angle-resolved XPS (ARXPS). The Ga-to-O ratio of the β-Ga_2_O_3_ surface layer significantly increased with surface depth and was close to the stoichiometric ratio of 2/3, indicating that gallium salts and other oxide contaminants may be distributed on the surface. Trace amounts of metal contaminants, such as Na, were present on the surface and were likely contributed by the slurry containing sodium hydroxide. Fig. 4 and 6 suggested that metal ions can penetrate the crystal surface through mechanical abrasion during CMP but not during static etching. To prevent metal pollution, the slurry formula must be optimised by selecting the appropriate organic alkali and a suitable complexing agent.^19,29–31^ An extremely thin metamorphic layer was adsorbed on the crystal surface treated through CMP. This layer is useful for removing surface crystal materials during CMP.

### 3.3 Polishing mechanisms

The chemical action mechanism of β-Ga_2_O_3_ is summarised as the formation of a metamorphic surface layer. Firstly, XPS data shown in Fig. 5 indicated that the chemical action mechanism underlying CMP can be used to describe the formation of the metamorphic layer. When Ga_2_O_3_ was subjected to CMP with alkali hydroxide under basic pH, reaction products that contain gallium salts, such as Ga(OH)_4_^−^, may leave a film on the crystal surface.^20^ The reaction that yields gallium salt is as follows:

1 Ga_2_O_3_(s) + 2OH^−^(aq) + 3H_2_O(l) → 2[Ga(OH)_4_]^−^(aq)

SEM data shown in Fig. 3 revealed that a dendritic pattern appeared on the β-Ga_2_O_3_ crystal surface after immersion in slurry containing sodium hydroxide. This pattern indicated that the surface corrosion of β-Ga_2_O_3_ is selective and that generation of new metamorphic layers is uneven. Rugged metamorphic layers could be uniformly removed through mechanical abrasion to yield ultrasmooth, nondamaged crystal surfaces.

A metamorphic layer formed on the surface of β-Ga_2_O_3_ through a chemical reaction during CMP under alkaline conditions. This layer was mainly composed of gallium salts. The adsorption of these salts on the β-Ga_2_O_3_ surface changed the physical and chemical properties of the crystal surface, such as hardness, brittleness, wettability and hydrolysability, thus improving the processing properties of the crystal. Therefore, the generation of a metamorphic layer influences material removal during CMP and mitigates cleavage during polishing.

## 4 Conclusions

β-Ga_2_O_3_ (100) surfaces were treated through MP, CMP and etching. The treated surfaces were then characterised through AFM, LSCM, SEM and XPS to reveal the effect of sodium hydroxide on ultraprecision polishing. The LSCM results showed that the cleavage of β-Ga_2_O_3_ hindered the processing of crystals to an ultrasmooth surface, and the apparent fragility of the crystal during MP resulted in the formation of surface micropits. SEM and XPS results proved that the formation of a metamorphic layer on the surface of β-Ga_2_O_3_ has a critical role in the removal of materials formed by the chemical reaction between Ga_2_O_3_ and sodium hydroxide during CMP. Ga–O bonds on the surface of β-Ga_2_O_3_ replaced cleaved Ga–OH bonds, leading to the formation of soluble gallium salts. The metamorphic layer can be easily removed during CMP to prevent surface damage. SEM results showed that dendritic patterns formed on the crystal surface after etching. These patterns indicated that β-Ga_2_O_3_ is nonuniformly and selectively corroded during chemical reaction due to. AFM indicated that an ultrasmooth surface with a low *R*_a_ of approximately 0.18 nm was obtained through CMP. In summary, the chemical reaction of OH^−^ on β-Ga_2_O_3_ may help aid the optimisation of slurry preparation for CMP.

## Conflicts of interest

There are no conflicts to declare.

## Supplementary Material
